# Combined didactic and scenario-based education improves the ability of intensive care unit staff to recognize delirium at the bedside

**DOI:** 10.1186/cc6793

**Published:** 2008-02-21

**Authors:** John W Devlin, Francois Marquis, Richard R Riker, Tracey Robbins, Erik Garpestad, Jeffrey J Fong, Dorothy Didomenico, Yoanna Skrobik

**Affiliations:** 1 School of Pharmacy, Northeastern University, 360 Huntington Avenue, Boston, MA 02118, USA; 2 Department of Pharmacy, Tufts-New England Medical Center, 750 Washington Street, Boston, MA 02111, USA; 3 Department of Critical Care Medicine, Maisoneuve-Rosemont Hospital, 5415 de l'Assomption, Montreal, QC H1T 2M4, Canada; 4 Department of Critical Care Medicine, Maine Medical Center, Portland, ME 04102, USA; 5 Division of Pulmonary, Critical Care and Sleep Medicine, Tufts-New England Medical Center, 750 Washington Street, Boston, MA 02111, USA; 6 Department of Nursing, Tufts-New England Medical Center, 750 Washington Street, Boston, MA 02111, USA

## Abstract

**Background:**

While nurses play a key role in identifying delirium, several authors have noted variability in their ability to recognize delirium. We sought to measure the impact of a simple educational intervention on the ability of intensive care unit (ICU) nurses to clinically identify delirium and to use a standardized delirium scale correctly.

**Methods:**

Fifty ICU nurses from two different hospitals (university medical and community teaching) evaluated an ICU patient for pain, level of sedation and presence of delirium before and after an educational intervention. The same patient was concomitantly, but independently, evaluated by a validated judge (ρ = 0.98) who acted as the reference standard in all cases. The education consisted of two script concordance case scenarios, a slide presentation regarding scale-based delirium assessment, and two further cases.

**Results:**

Nurses' clinical recognition of delirium was poor in the before-education period as only 24% of nurses reported the presence or absence of delirium and only 16% were correct compared with the judge. After education, the number of nurses able to evaluate delirium using any scale (12% vs 82%, *P *< 0.0005) and use it correctly (8% vs 62%, *P *< 0.0005) increased significantly. While judge-nurse agreement (Spearman ρ) for the presence of delirium was relatively high for both the before-education period (*r *= 0.74, *P *= 0.262) and after-education period (*r *= 0.71, *P *< 0.0005), the low number of nurses evaluating delirium before education lead to statistical significance only after education. Education did not alter nurses' self-reported evaluation of delirium (before 76% vs after 100%, *P *= 0.125).

**Conclusion:**

A simple composite educational intervention incorporating script concordance theory improves the capacity for ICU nurses to screen for delirium nearly as well as experts. Self-reporting by nurses of completion of delirium screening may not constitute an adequate quality assurance process.

## Introduction

Delirium in the intensive care unit (ICU) is associated with an increased mortality and a longer ICU and hospital stay [1-3]. Practice guidelines for sedation and analgesia in the ICU recommend that patients be routinely screened for delirium using a validated assessment tool [4]. Given the fluctuating nature of delirium symptoms, the bedside nurse is the ICU caregiver best suited to screen for delirium [5-8]. While education plays a key role in boosting delirium screening efforts by ICU nurses, the optimal pedagogical strategy to educate clinicians regarding delirium assessment is currently unclear and several authors have pointed out the limitations of standard delirium teaching methods [6,9,10]. Also, although traditional didactic lectures are proven to train nurses to individually apply sedation and pain scales at the bedside, these assessment tools only evaluate one dimension of an ICU patient's symptoms [11,12]. In addition, traditional didactic approaches such as classroom instruction are notoriously limited in modifying clinician behavior [13,14].

Pain, anxiety and delirium are common in the ICU and may sometimes be difficult to separate due to overlapping and confounding symptoms [4,8,15]. Critical care clinicians therefore require clinical reasoning to optimally evaluate patients for the presence of each [9,11,16]. Script concordance, adapted from cognitive psychological theory, integrates clinical reasoning and experience, and has been shown to be of benefit in other areas of clinical practice where decision-making is complex [17-21]. As often occurs in real life, incomplete information is given with script concordance tools, requiring the use of reasoning skills and past experience to make a final judgment. This approach may prove especially effective for assessing complex clinical conditions such as delirium [17-21]. This prospective study measured the impact of an educational intervention, incorporating a didactic presentation and before-and-after case scenarios utilizing script concordance methodology, on the ability of nurses from the ICUs at two different types of hospitals to clinically identify delirium and use a standardized delirium scale correctly.

## Materials and methods

ICU nurses from two institutions participated in the study: from the 10-bed medical ICU at Tufts-New England Medical Center, a 450-bed academic medical center in Boston, MA, USA; and the 32-bed mixed medical-surgical ICU at Maine Medical Center, a 599-bed community teaching hospital in Portland, ME, USA. Both ICUs have used the Sedation-Agitation Scale and a 10-point numeric pain scale (each validated for use in the ICU) for patient assessment for longer than 5 years, and at the time of the study neither ICU was using a delirium scale routinely for patient assessment [22]. The present study was approved by institutional review boards at both centers. While each nurse provided informed consent prior to participation in the study, the need for consent from patients was waived.

A number of delirium assessment tools are available for use in the ICU [8,23,24]. After reviewing these options, we selected the Intensive Care Delirium Screening Checklist (ICDSC) because it evaluates patients in real time over the entire nursing shift, provides a graded scoring assessment rather than a dichotomous approach, and is favored by the onsite nurse educators [23,25]. The ICDSC has been validated in several studies in multiple countries for medical and surgical ICU patients, and has a sensitivity of 99% when compared with psychiatrist evaluation using Diagnostic and Statistical Manual of Mental Disorders IV criteria and a reliability >90% [23,25,26].

The study personnel (one intensivist, two critical care nurses, one critical care pharmacist) were trained by one of the researchers (YS), who developed and first validated the ICDSC. This education included an initial 2-day training session including didactic practice, question-and-answer practice, and bedside assessment practice at both centers. Three months later, a second meeting was designed to confirm the reliability of our researchers compared with the expert assessment of this same ICDSC designer. If the researcher was able to attain a reliability superior to 90% in the ICSDC patient assessment, they were deemed to be certified as reference standard judges [23]. These judges (two at each center) could then evaluate nursing performance before and after teaching, in real time, at each center. Using an ICDSC worksheet (Additional file 1) jointly developed by RRR and JWD, each evaluator concomitantly, but independently, and sequentially evaluated five different critically ill patients' level of sedation (using the Sedation-Agitation Scale) and whether delirium (using the ICDSC) was present [22]. The rater's assessment of pain intensity was documented using a 0–10 numeric rating scale. After each patient in this practice set, the members of the group discussed their assessments. After this training session, a test-round evaluation of 10 different patient evaluations was conducted to assess reliability compared with the ICDSC designer. The correlation between the ICDSC designer and our research judges was excellent, with adjudication for level of pain and sedation having Spearman ρ = 1.0 and each clinical characteristic featured in the ICDSC having Spearman ρ ≥ 0.92. Single evaluator judges at each site were thus considered equivalent reference standards for all further nursing performance assessments to occur at each center.

After this judge reliability was confirmed, a convenience sample of 50 critical care nurse volunteers (25 nurses at each center) completed all three components of the study during one shift on the same day: a before-education bedside assessment observed by a validated judge, a three-step educational intervention, and an after-educational bedside evaluation of a different patient observed by a validated judge. Based on a bilateral testing model, we estimated 50 nursing subjects would be required to detect a 25% improvement in the appropriate use of delirium scales with a power of 80% and a final *P *value of 0.05.

Patients evaluated in the context of the study were systematically selected by one of the investigators for evaluation by moving sequentially from the lowest to highest bed number in the study ICU at each institution. Patients admitted with a primary neurologic disorder (for example, stroke) or alcohol withdrawal were excluded. Neither routine patient care nor the administration of sedation, analgesia or psychotropic therapy, as prescribed by the patient's primary physician, were altered. One hundred different patients were used for the pre-study and post-study evaluations, and the nurses being tested were not caring for the patient they assessed that day. At the beginning of the before-education bedside nursing observation, each nurse was specifically asked to determine (clinically) whether the assessed patient was delirious or not, and to document the observation on the documentation worksheet. This instruction was explicit and independent of the request to use a validated delirium assessment scale, in order to capture clinical assessment skills independent from those related to use of a validated scale. The nursing subject was then (during the same evaluation) instructed to apply any scale or other screening method with which they may be familiar. The trained bedside judge and nursing subject evaluated the patient simultaneously but independently.

Each nursing evaluation was adjudicated as follows: whether the dimension (delirium) was evaluated at all, whether the dimension was evaluated with an appropriate scale (either the ICDSC or the Confusion Assessment Method for the Intensive Care Unit), and whether the scale was used correctly. In all instances, both the adjudicator and subject nurse could communicate with the patient's primary nurse to garner information pertaining to any delirium symptom that was temporally related (for example, symptom fluctuation) or occurred during the past shift (for example, reaction to visit by a family member, sleep or device removal). Given that pain may cause agitation (one of the eight ICDSC items) and that the ICDSC cannot be used when a patient is heavily sedated (Sedation-Agitation Scale ≤ 2), both the nurse and adjudicator first evaluated each patient's level of pain intensity using a 0–10 numeric rating scale and then sedation using the Sedation-Agitation Scale prior to evaluating delirium items [22]. Results were descriptively compared between the nurse subject and the gold standard judge for those evaluations where a scale was used, and in those instances for whether the scale was used correctly.

The educational intervention consisted of two sets of two clinical-based scenarios written based on script concordance theory. These sandwiched a more conventional didactic slide presentation about delirium evaluation, which included the use of pain, sedation and delirium scales. The four different ICU patient scenarios (Additional file 2) and accompanying questions were previously developed and validated by a focus group at the University of Montreal's Maisonneuve-Rosemont Hospital. That development/validation group included two experienced intensivists (one medical, one anesthetist), two experienced (>15 years of practice) critical care nurses, one critical care research nurse, two critical care nurse specialist teachers, and a clinical ICU pharmacist. Consistent with script concordance theory, each patient scenario contained uncertain or insufficient information about delirium symptoms, and incorporated pain and sedation levels to better reflect the reality of clinical practice.

These scenarios were included as part of the educational component, not as part of the assessment, to improve the ability of nurses to use the ICDSC to detect delirium and to foster information-gathering and clinical reasoning. The nurses were asked to reflect on whether the patient in the scenario had delirium, and which of the described clinical features supported this premise. The order of the before and after scenarios was randomly assigned to the evaluated nurses. The didactic presentation consisted of a 20-slide presentation that reviewed the basics of pain, sedation, and delirium evaluation lasting 30–45 minutes. The presentation was conducted in an ICU conference room to groups of at least two nurses during the same shift as the bedside evaluations. The same presentation was used at each site and was developed by YS, RRR and JWD.

The impact of the educational intervention was measured by evaluating both the nurses' ability to clinically identify delirium as well as their ability to use a standardized delirium scale correctly. Paired-samples tests for binary results (McNemar) were used to compare the bedside evaluations before and after the pedagogical intervention, and the Spearman ρ value was used to measure reliability between the judges and the ICDSC designer and between the judge and the nurse. All statistical analysis was performed using the SPSS 14.0 (SPSS Inc., Chicago, IL, USA) statistical package, and *P *≤ 0.05 was considered statistically significant.

## Results

The 50 nurse subjects had an average of 14.4 ± 9.2 years of experience. The 100 patients had an average age of 52 ± 16 years and an average Acute Physiology and Chronic Health Evaluation II score at ICU admission of 19.6 ± 6.7 [27]. Clinical recognition of delirium was poor in the before-education group, as only 24% of the nurses reported the presence or absence of delirium and only 16% were correct compared with the judge. There was a sevenfold increase in the number of nurses who used a validated delirium screening tool (12% vs 82%, *P *< 0.0005) and used it correctly (8% vs 62%, *P *< 0.0005) after the educational intervention, reflecting a significant objective performance improvement (Tables 1 and 2). While the judge-nurse agreement for the presence of delirium was relativelyhigh for both the before-education (r = 0.738, p = 0.262) and after-education (r = 0.714, *P *< 0.0005) periods, the low number of nurses evaluating delirium prior to education lead to statistical significance in only the after-education period.

**Table 1:** Nursing assessment of delirium, pain, and sedation before and after the educational intervention

	Before education (*n *= 50)	After education (*n *= 50)	After education (%):before education (%) ratio	*P *value
Delirium				
Presence or absence of delirium reported by nurse (%)	24	100	4.3	<0.0005
Assessed using a scale (%)	12	82	7.5	<0.0005
Correct use of the scale (%)	8	62	7.5	<0.0005
Self-report assessed (%)	74	100	-	0.125
Pain^a^				
Assessed using a scale (%)	46	62	1.4	
Correct use of the scale (%)	20	55	2.8	
Self-report assessed (%)	54	66	-	
Sedation^a^				
Assessed using a scale (%)	83	98	1.2	
Correct use of the scale (%)	76	98	1.3	
Self-report assessed (%)	92	98	-	

^a^These comparisons are purely descriptive and exploratory.

**Table 2:** Agreement between judges and nurses before and after the educational intervention

	Before education (*n *= 50)			After education (*n *= 50)		
	
	Spearman ρ	*P *value	Completed by nurses (%)	Spearman ρ	*P *value	Completed by nurses (%)
Presence of delirium	0.74	0.262	8	0.71	<0.0005	62
Presence of pain	0.94	<0.0005	20	0.34	0.076	56
Presence of sedation	0.89	<0.0005	76	0.99	<0.005	98

Paradoxically, the percentage of nurses self-reporting adequate patient delirium assessments did not change significantly after the educational intervention (before 76% vs after 100%, *P *= 0.125), suggesting discordance between self-assessment and objective measures of delirium assessment. Strictly descriptive comparisons of pain and sedation assessments before and after the educational intervention are provided in Tables 1 and 2. Compared with the significant increase for use of the delirium screening tool, a smaller increase was noted by nurses who used a validated sedation (1.18) or pain (1.35) assessment tool and by nurses who used a sedation (1.29) or pain (2.75) assessment tool correctly.

## Discussion

Screening for delirium in the ICU is probably most effective if clinicians are trained in the use of standardized tools, since the ability to identify delirium in the ICU improves when a validated delirium assessment scale is used [6,28]. This is the first study of ICU delirium assessment to measure the effect of an educational intervention encompassing both didactic and clinical-reasoning-based teaching methods [29]. Integrating a clinical-reasoning-based pedagogical approach such as script concordance in our educational efforts matches the day-to-day experiences of most ICU nurses where clinical confounders and insufficient information are common [17-21]. The combination of a didactic lecture and clinical-reasoning-based case scenarios improved the delirium assessment performance in our cohort.

During the educational intervention, all aspects related to the assessment of patient comfort (that is, pain, sedation and delirium, and their respective rating scales) were integrated into the didactic lecture presentation. Delirium evaluation with a script-concordance-based scenario approach was emphasized to all nurses participating in the study. Although assessment of delirium, pain, and sedation all increased significantly after the educational program, there was a much greater increase in delirium assessment. This may be explained by the fact that both pain and sedation assessment using validated scales had been formally implemented in both units, by the greater emphasis on delirium as the focus of the educational intervention, and by the challenges of evaluating pain in the ICU. These findings also suggest that educational methods combining didactic presentations and script-concordance-based questions are less effective in teaching pain or sedation assessment than delirium assessment. The nearly eightfold increase in the proportion of nurses who used the ICDSC correctly, however, with performance nearly matching the experts on their first try after a brief educational intervention, suggests a benefit from incorporating a script concordance approach in delirium education initiatives. Our study also suggests a limited ability of ICU nurses to evaluate their own performance with regard to patient assessments for delirium (regardless of the use of a validated tool).

The strengths of our investigation include its rigorous pedagogical methodology and its broad applicability to varying types of medical institutions (that is, both academic medical centers and community teaching hospitals) and varying patient populations (that is, both medical and surgical populations). Potential weaknesses include the very brief time frame over which evaluation occurred, which precludes the ability to confirm the sustainability of our educational efforts. While the study was powered to evaluate nurses' ability to identify delirium and use a delirium scale correctly, it was not adequately powered to evaluate nurses' assessment of pain or sedation and thus all observations regarding pain and sedation assessment should be considered exploratory. The relationship between nurses' perceptions and knowledge regarding delirium prior to the study and their performance in the study was not evaluated. In addition, nurses with prior experience using a validated delirium screening tool were not excluded from the study. Given the fact that the nurses were the subjects in our study, and not the patients who were being evaluated, we collected limited demographic data. Finally, it is possible that some of the improvement that was observed after education was simply a result of the clinical experience regarding delirium assessment gained through completion of the before-education patient assessment.

Our study highlights a number of areas for future research, such as determining the relative merit of specific pedagogical interventions on the ability of clinicians to identify delirium (that is, bedside teaching vs didactic classroom vs script concordance case scenarios) and evaluating the sustainability of the benefit we observed given that improvements associated with clinician education may be only temporary without further reinforcement and review [30,31]. The impact of follow-up educational sessions – which could be made up of both one-to-one and group discussions, and that have been shown to be of benefit in areas outside the ICU – should be evaluated [10]. The impact of other strategies such as ICU reorganization and changes to the care process that have been recently shown to improve patient outcomes related to sedation need to be evaluated for delirium [32]. The low recognition of pain by nurses in our study reflects work by Puntillo and others, and warrants a larger study powered to address potentially useful pedagogic interventions in this important area [9]. The impact of delirium assessment on patient outcomes is unknown [33]. Future studies need to evaluate simple strategies that can be employed in everyday clinical practice to measure the quality of the delirium assessment that nurses employ.

## Conclusion

A simple composite educational intervention incorporating script concordance theory rapidly improves the capacity for ICU nurses to perform delirium assessment in a standardized fashion without a detrimental effect on accuracy. This study also suggests that self-reporting of delirium screening may not constitute an adequate quality assurance process, and therefore that a standardized approach to identifying delirium in ICU patients should be incorporated in the education of critical care nurses. Finally, educational initiatives focused on improving the ability of bedside clinicians to assess delirium are at least as important as those for the assessment of pain and sedation, and should be part of any ICU patient improvement effort.

## Key messages

• Screening for delirium in the ICU is probably most effective if clinicians are trained in the use of standardized tools, since the ability to identify delirium in the ICU improves when a validated delirium assessment scale is used.

• Use of the combination of a didactic lecture and clinical-reasoning-based case scenarios for delirium education rapidly improves the capacity for ICU nurses to perform delirium assessment in a standardized fashion without detrimental effect on accuracy.

• Self-reporting of delirium screening may not constitute an adequate quality assurance process.

• Implementation and evaluation of educational initiatives addressing the ability of bedside clinicians to assess delirium are at least as important as those for the assessment of pain and sedation and should be part of any ICU patient improvement effort.

## Abbreviations

ICDSC = Intensive Care Delirium Screening Checklist; ICU = intensive care unit.

## Competing interests

The authors declare that they have no competing interests.

## Authors' contributions

JWD was responsible for the concept, acquisition and interpretation of data, manuscript preparation, and final manuscript approval. FM was responsible for the concept, analysis and interpretation of data, manuscript preparation, and final manuscript approval. RRR was responsible for the concept, acquisition and interpretation of data, manuscript preparation, and final manuscript approval. TR, EG, JJF and DD were responsible for the acquisition of data, manuscript preparation, and final manuscript approval. YS was responsible for the concept, acquisition, analysis and interpretation of data, manuscript preparation, and final manuscript approval.

## Supplementary Material

Additional file 1 containing a table that presents the Intensive Care Delirium Screening Checklist Worksheet. Click here for file

Additional file 2 containing descriptions of the four different test cases. Click here for file
