# Supplementary material for: Combined didactic and scenario-based education improves the ability of intensive care unit staff to recognize delirium at the bedside
Source: Crit Care. 2008 Feb 21;12(1):R19. doi: 10.1186/cc6793 (PMC2374631; doi:10.1186/cc6793)
Supplement: Additional file 1 — containing a table that presents the Intensive Care Delirium Screening Checklist Worksheet. [file cc6793-S1.doc]

Appendix #1 Intensive Care Delirium Screening Checklist Worksheet

| **Date** |  |  |  |  |  |
| --- | --- | --- | --- | --- | --- |
| **Time** |  |  |  |  |  |
| **1. Altered level of consciousness** Choose **ONE** from A-E.  Note: May need to reassess patient if recent administration of sedation therapy |  |  |  |  |  |
| A. Exaggerated response to normal stimulation Riker/SAS = 5, 6, or 7  Score 1 point |  |  |  |  |  |
| B. Normal wakefulness Riker/SAS = 4 Score 0 points |  |  |  |  |  |
| C. Response to mild or moderate stimulation Riker/SAS = 3 Score 1 point  (follows commands) **Score 0 if LOC related to recent sedation/analgesia** |  |  |  |  |  |
| D. Response only to intense and repeated stimulation (e.g. loud voice and pain)  SAS = 2 ****Stop assessment** |  | - | - | - | - |
| E. No response SAS = 1 ****Stop assessment** |  | - | - | - | - |
| **2. Inattention** Score 1 point for any of the following abnormalities:  A. Difficulty in following commands OR  B. Easily distracted by external stimuli OR  C. Difficulty in shifting focus  **Does the patient follow you with their eyes?** |  |  |  |  |  |
| **3. Disorientation** Score 1 point for any one obvious abnormality:  A. Mistake in either time, place or person  **Does the patient recognize ICU caregivers who have cared for him/her and not recognize those that have not? What kind of place are you in? (list examples)** |  |  |  |  |  |
| **4.** **Hallucinations or Delusions** Score 1 point for either :  A. Equivocal evidence of hallucinations or a behavior due to hallucinations  (Hallucination = perception of something that is not there with NO stimulus) OR  B. Delusions or gross impairment of reality testing  (Delusion = false belief that is fixed/unchanging)  **Any hallucinations now or over past 24 hrs? Are you afraid of the people or things around you? [fear that is inappropriate to clinical situation]** |  |  |  |  |  |
| **5.** **Psychomotor Agitation or Retardation** Score 1 point for either:  A. Hyperactivity requiring the use of additional sedative drugs or restraints in  order to control potential danger (e.g. pulling IV lines out or hitting staff) OR  B. Hypoactive or clinically noticeable psychomotor slowing or retardation  **Based on documentation and observation over shift by primary caregiver** |  |  |  |  |  |
| **6. Inappropriate Speech or Mood** Score 1 point for either:  A. Inappropriate, disorganized or incoherent speech OR  B. Inappropriate mood related to events or situation  **Is the patient apathetic to current clinical situation (ie. lack of emotion)?**  **Any gross abnormalities in speech or mood? Is patient inappropriatelydemanding?** |  |  |  |  |  |
| **7. Sleep/Wake Cycle Disturbance** Score 1 point for:  A. Sleeping less than four hours at night OR  B. Waking frequently at night (do not include wakefulness initiated by medical  staff or loud environment) OR  C. Sleep ≥ 4 hours during day **Based on primary caregiver assessment** |  |  |  |  |  |
| 8. **Symptom Fluctuation** Score 1 point for:  fluctuation of any of the above items (ie. 1 – 7) over 24 hours (e.g. from one  shift to another) **Based on primary caregiver assessment** |  |  |  |  |  |
| **TOTAL ICSDC SCORE (Add 1 – 8)** |  |  |  |  |  |

**A total ICSDC Score ≥ 4 has a 99% sensitivity correlation for a psychiatric diagnosis of delirium** Source: Bergeron N et al. Intensive Care Med 2001; 27:869-64 **Revised July 22 2005**
