# Supplementary material for: Combined didactic and scenario-based education improves the ability of intensive care unit staff to recognize delirium at the bedside
Source: Crit Care. 2008 Feb 21;12(1):R19. doi: 10.1186/cc6793 (PMC2374631; doi:10.1186/cc6793)
Supplement: Additional file 2 — containing descriptions of the four different test cases. [file cc6793-S2.doc]

Appendix #2: Testing Cases

The following are descriptions of four different ICU patient scenarios. We would like you to evaluate each patient’s level of pain, sedation, and delirium using the specific questions that follow each case. Please answer the questions to the best of your ability without input from your colleagues

*Clinical scenario #1*

A 78-year-old patient was admitted 48 hours ago with *C. difficile* colitis and hypotension caused by her dehydration and sepsis. She underwent a colectomy yesterday. She is transferred to your care in the SICU in the evening. You note in her chart that she is diabetic. Her colitis occurred when antibiotics were administered for a urinary infection while she was hospitalized in a nearby hospital for a foot fracture.

When you approach the patient, she does not move much. Her eyes are squeezed shut. She does not respond to your questions and does not open her eyes when you speak to her. When you begin your evaluation and are about to reposition her in bed, she groans and says “Oy, oy, oy.” She also seems to be calling on God and the Virgin Mary between saying this, but she is not speaking clearly. When you try to move her, she clenches her fists.

a. Is this patient in pain? Please provide justification to support your answer that may include the use of a bedside assessment scale/tool

b. What is her level of sedation? Please provide justification to support your answer that may include the use of a bedside assessment scale/tool

c. Is she delirious? Please provide justification to support your answer that may include the use of a bedside assessment scale/tool

While you are caring for her, her daughter arrives. She explains that her mother has a unique way of expressing herself, with the exclamation “oy, oy, oy” being part of her basic vocabulary. She tells you that her mother has a long history of anxiety for which she has been taking benzodiazepines, and has been drinking alcohol for twenty years.

Does your evaluation change? If so, how; if not, why not?

Pain: Change in previous evaluation? Yes No (choose one)

If yes, please provide rationale to support your answer

Sedation: Change in previous evaluation? Yes No (choose one)

If yes, please provide rationale to support your answer

Delirium: Change in previous evaluation? Yes No (choose one)

If yes, please provide rationale to support your answer

What additional information would you like to obtain to be better able to assess this patient’s level of pain, sedation and/or delirium?

*Clinical Scenario #2*

Mr. G., 47 years old, is schizophrenic and has been living in an institution for over 20 years.

He was admitted to intensive care two days ago, for respiratory decompensation due to pneumonia. Upon arrival, Mr. G. was calm, slightly slow to react, and unable to name the hospital where he had been transferred or the date. He was not intubated. Around 6:00 PM, the nurse charted the patient as being drowsy but easily awakened, and documented that he did not always answer questions. The nurse also noted that Mr. G. did not seem to be suffering, as he shook his head “no” when she asked him if he was in pain. She recorded that his state of consciousness and attentiveness were altered, and that he showed psychomotor retardation and space-time disorientation.

Upon his arrival, the day nurse had described Mr. G. as calm and alert, not in pain, slow to react, and disoriented in space and time. She reported that it was difficult to keep his attention and that he repeated the same questions over and over. Around 9:00 AM, a desaturation episode required the application of a positive pressure mask, which Mr. G. tolerated with great difficulty. He became more and more agitated, constantly moving around in his bed, trying to remove the mask, and not listening when spoken to. The nurse also noted that he startled every time someone approached the bed. The physician was called; when he arrived 30 minutes later, he noted that the patient was somewhat agitated but was cooperative and answered questions appropriately, though he remained disoriented in time and space. Mr. G. said that he was thirsty and he wanted to smoke. The physician prescribed Ativan p.r.n., and said to remove the mask five minutes per hour if saturation remained above 90%.

a. Is this patient in pain? Please provide justification to support your answer that may include the use of a bedside assessment scale/tool

b. What is his level of sedation? Please provide justification to support your answer that may include the use of a bedside assessment scale/tool

c. Is he delirious? Please provide justification to support your answer that may include the use of a bedside assessment scale/tool

During the first night, the patient becomes incoherent and begins to groan in bed. You cannot elicit a response from him; he does not answer your questions. His heart rate, previously 90-95, has increased to 120. He remains afebrile. His facial muscles are clenched.

Please reassess this patient based on these new clinical findings:

a. Is this patient in pain? Please provide justification to support your answer that may include the use of a bedside assessment scale/tool

b. What is his level of sedation? Please provide justification to support your answer that may include the use of a bedside assessment scale/tool

c. Is he delirious? Please provide justification to support your answer that may include the use of a bedside assessment scale/tool

*Clinical scenario #3*

Mrs. X. was admitted to the intensive care unit three days ago secondary to a drug overdose.

She was comatose, unresponsive, calm and without spontaneous movements for two days. She was intubated upon arrival, but she was not given sedation to allow for re-evaluations of her neurological status.

Today, the patient is agitated and is biting her endotracheal tube. She has even had to be restrained because she has tried to extubate herself several times. She does not make eye contact or respond to orders. The ventilator’s alarm is constantly ringing.

a. Is this patient in pain? Please provide justification to support your answer that may include the use of a bedside assessment scale/tool

b. What is her level of sedation? Please provide justification to support your answer that may include the use of a bedside assessment scale/tool

c. Is she delirious? Please provide justification to support your answer that may include the use of a bedside assessment scale/tool

d. Should sedative medication(s) be administered? Yes No (choose one)

Please provide justification for your answer.

# Clinical scenario #4

(as narrated by the caregiver nurse)

A 60-year-old man is admitted to the coronary unit after a myocardial infarction. The patient has significant visual impairment and can see only shadows.

The previous nurse noted that the patient is spitting up his pills and reacts negatively when he is approached (e.g. to collect vitals). He is very verbally aggressive. He slept for only part of the night.

When I enter the room, I introduce myself while remaining far from his bed. I explain that I will be his nurse today and that I have to give him his medication. Initially he refuses. I ask why; he is capable of reasoning and is not confused, and finally lets me approach and he takes his medication. I note that he startles easily and holds his arm out to defend himself when notices someone approaching him. He is agitated and aggressive but is not pulling on his tubes or attempting to get up.

He is not confused, but is easily angered. He lashes out at the air with his arms, and resists being touched particularly if he is not warned beforehand. When he becomes angry, his attention becomes altered and he stops listening when spoken to. He is calm when alone in the room.

As time passes, I realize that the patient is afraid because he cannot see well. When he is told what is being done to him, he is much less aggressive, and even becomes cooperative. He quickly recognizes my voice and calms down when I speak to him. If another person approaches, he shows the same pattern as before – he is fearful but can be calmed down once the person greets him.

a. Is this patient in pain? Please provide justification to support your answer that may include the use of a bedside assessment scale/tool

b. What is his level of sedation? Please provide justification to support your answer that may include the use of a bedside assessment scale/tool

c. Is he delirious? Please provide justification to support your answer that may include the use of a bedside assessment scale/tool
